# Transcriptome-Wide Detection of Intron/Exon Definition in the Endogenous Pre-mRNA Transcripts of Mammalian Cells and Its Regulation by Depolarization

**DOI:** 10.3390/ijms231710157

**Published:** 2022-09-05

**Authors:** Ling Liu, Urmi Das, Samuel Ogunsola, Jiuyong Xie

**Affiliations:** Department of Physiology & Pathophysiology, Max Rady College of Medicine, Rady Faculty of Health Sciences, University of Manitoba, Winnipeg, MB R3E 0J9, Canada

**Keywords:** alternative splicing, reads at the intron start/end (RISE), splice site-pairing, exon definition, intron definition, depolarization

## Abstract

Pairing of splice sites across an intron or exon is the central point of intron or exon definition in pre-mRNA splicing with the latter mode proposed for most mammalian exons. However, transcriptome-wide pairing within endogenous transcripts has not been examined for the prevalence of each mode in mammalian cells. Here we report such pairings in rat GH3 pituitary cells by measuring the relative abundance of nuclear RNA-Seq reads at the intron start or end (RISE). Interestingly, RISE indexes are positively correlated between 5′ and 3′ splice sites specifically across introns or exons but inversely correlated with the usage of adjacent exons. Moreover, the ratios between the paired indexes were globally modulated by depolarization, which was disruptible by 5-aza-Cytidine. The nucleotide matrices of the RISE-positive splice sites deviate significantly from the rat consensus, and short introns or exons are enriched with the cross-intron or -exon RISE pairs, respectively. Functionally, the RISE-positive genes cluster for basic cellular processes including RNA binding/splicing, or more specifically, hormone production if regulated by depolarization. Together, the RISE analysis identified the transcriptome-wide regulation of either intron or exon definition between weak splice sites of short introns/exons in mammalian cells. The analysis also provides a way to further track the splicing intermediates and intron/exon definition during the dynamic regulation of alternative splicing by extracellular factors.

## 1. Introduction

Pre-mRNA splicing occurs between pairing splice sites across an intron (intron definition) or exon (exon definition). The former is likely the preferred way of splicing in species such as drosophila with mostly short introns, and the latter in vertebrates with relatively short exons among long introns [1,2,3,4]. Both scenarios are supported by splice site cross-talks evident by the impact of one splice site defects on the other site across an intron or exon in the genetic or biochemical analysis [1,5,6,7], or global RNA-Seq analysis of pre-mRNA intermediates in association with intron/exon lengths [8]. The splicing intermediates provide targets for monitoring the splice site usage of endogenous pre-mRNA transcripts [8,9,10,11]. However, transcriptome-wide pairings of splice sites across introns or exons have not been reported. 

Our interest in the splice site pairing of the intermediates was prompted by the depolarization-regulated splicing of the STREX (stress hormone axis-regulated exon) in pituitary cells, a model exon for studying activity-dependent splicing [12,13,14,15,16]. The regulation is through calcium signaling and hnRNP L binding to a CaMKIV-responsive RNA element (CaRRE) at the upstream 3′ splice site of STREX [14,15]. However, it remains unclear whether the same site is also regulated in the endogenous pre-mRNA transcripts and/or whether it is controlled in pair with the upstream (intron definition) or downstream (exon definition) 5′ splice site by depolarization. Such questions could be examined by measuring the splicing intermediates using RNA-Seq analysis. The analysis would also allow tracking in future studies of the dynamic regulation process and further understanding of its molecular mechanisms, as part of the gene expression control for adaptation, synapse maturation, or hormone production by depolarization [12,13,15,17,18,19,20]. 

Here, we use RNA-Seq of nuclear RNA (rather than using total RNA, where the abundant, fully spliced cytoplasmic mRNA transcripts coexist with pre-mRNA intermediates limiting the reads coverage for the latter), to measure the ratio of reads at the intron start or end over exon reads (index), the global relationship between the indexes of splice sites across introns or exons and their regulation by membrane depolarization. This detected the transcriptome-wide pairing of indexes of 5′ and 3′ splice sites specifically across introns or exons including STREX in the transcriptome, and regulation of the pairing by depolarization. 

## 2. Results

### 2.1. Nuclear RNA Sequencing Reveals Accumulation of Splice Site-Specific Reads at the Intron Start or End of Alternative Exons

To track the nuclear pre-mRNA intermediates in the splicing regulation of STREX and other alternative exons by membrane depolarization, we made use of the RNA-Seq samples from nuclear, cytoplasmic, or whole cell lysates of rat GH3 pituitary cells (see Materials and Methods for more details). We analyzed the uniquely mapped read pairs using DEXSeq or edgeR, for the Ensembl-annotated (spliced) RNA transcripts or their intron regions.

**Figure 1:**
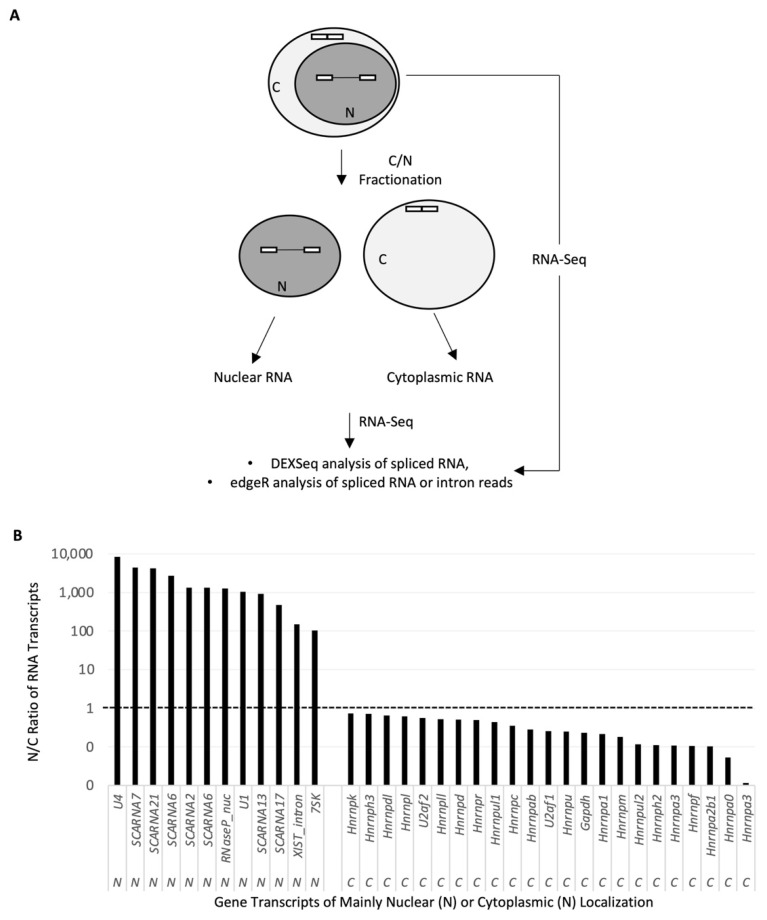
RNA-Seq samples and workflow of analysis. (**A**) Diagram of the nucleo-cytoplasmic fractionation and subsequent analyses of the RNA-Seq data of RNA from different fractions. (**B**) Efficiency of the fractionation based on the nucleocytoplasmic (N/C) distribution ratios of the subcellular location-known RNA transcripts. Ratio determined by the edgeR analysis of the RNA-Seq data of nuclear versus cytoplasmic RNA fractions of KCl-depolarized samples.

The nuclear RNA fraction was highly enriched with known nuclear RNA transcripts by edgeR analysis of their relative levels over those in the cytoplasmic fractions (Figure 1). In particular, the *U4* and *U1* snRNAs, Small Cajal body-specific RNAs *ScaRNA2, 6, 7, 13, 17,* and *21*, *RNase-P* nuclear RNA, *Xist*-intron RNA, and *7SK* RNA were about 100 to 10,000 times enriched, consistent with their nuclear localization [21,22,23,24,25]. In contrast, the spliced mRNA transcripts of the *Hnrnp*, *U2af,* and *Gapdh* genes all had an N/C ratio of less than 1, consistent with their mainly cytoplasmic localization. This verified the specificity of the nuclear RNA samples by our nucleo-cytoplasmic RNA fractionation method.

**Figure 2:**
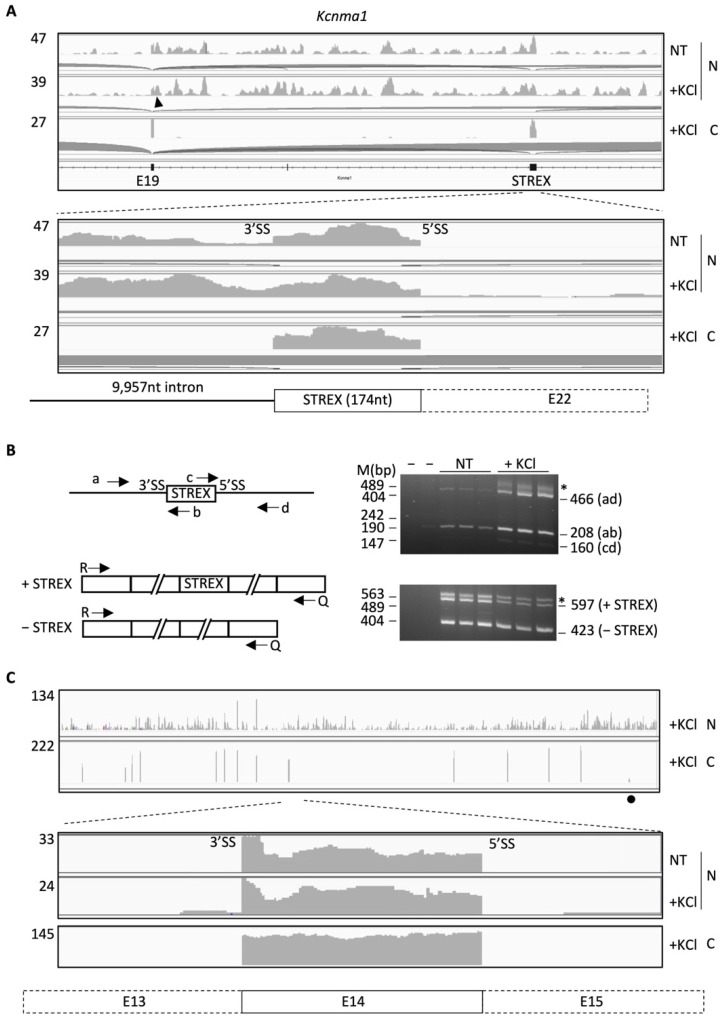
Differential distribution of RNA-Seq reads at the intron start/end (splice site) of nuclear RNA transcripts. Shown are the pre-mRNA regions containing the alternative STREX exon in IGV views (**A**), RT-PCR products (**B**) or the constitutive exon 14 (**C**) of the *Kcnma1* gene on chromosome 15 (Chr15: 911–927 kb) of the rat GH_3_ pituitary cells without (NT) or with KCl treatment. Under each enlarged view are deduced major patterns of spliced intermediates of the pre-mRNA transcripts in the NT samples. The curves extending from the exon start/end denote splice junctions. To the left of each IGV view is the upper limit of read counts for each sample. Arrowhead: upstream 5′ splice site also with read peaks. N: nuclear fraction, C: cytoplasmic fraction. Black dot: STREX. In (**B**), the RT-PCR was from total RNA of NT or KCl-treated cells for both pre-mRNA and spliced products. Arrowheads: PCR primers. Diagrams not to scale. Note that the R/Q primers for the spliced products span multiple exons/introns of the gene. The left two lanes are negative controls, PCR without cDNA and RT (KCl-treated) without reverse transcriptase, respectively. The RT control lane has a 208 bp band but is much weaker than the sample lanes (~5% of the band in the KCl-treated samples). *: heteroduplexes, with half of the band intensities added to each spliced variant for product calculations. M: DNA markers. Numbers to the right of the gels are the expected sizes of the PCR products.

Manual examination of the STREX exon and its flanking introns in the integrated genomics viewer (IGV [26]) identified intron reads at its upstream 3′ but not downstream 5′ splice site (SS) in the nuclear RNA-Seq samples (Figure 2A, non-treated, NT). Upon KCl-treatment (50 mM), the 3′SS read abundance (relative to the 5′ end of the exon) increased from about 30% to 67% on average (*p* < 0.001, hypergeometric density test) with little increase at the downstream 5′SS. The prominent increase of the upstream 3′SS reads is consistent with its increase in RT-PCR from the pre-mRNA at about five times over that of the downstream 5′SS (Note: band intensity normalized with product lengths, *p* < 0.01, Figure 2B), and is in contrast to the decreased STREX usage in the KCl-treated samples (from 31% to 21% on average, *p* < 0.005) similarly as in previous reports [13,27,28]. There were neither such reads in the cytoplasmic samples where the STREX exon reads were abundant (Figure 2A) nor the comparable level of reads around the constitutive exon 14 in the nuclear samples (Figure 2C). Moreover, the 5′ splice site in the upstream intron of STREX accumulated reads as well in the nuclear RNA samples (Figure 2A). Thus, splicing of the STREX upstream intron was specifically inhibited in some pre-mRNA transcripts in the NT and further in the KCl-treated nuclear RNA samples. This is consistent with the inhibition of the upstream 3′ splice site by depolarization and calcium signaling in minigene splicing reporter assays [13,14,15] and supports mainly delayed intron rather than exon definition in the depolarization-regulation of the STREX exon.

Similar splice site-specific accumulation of reads was also observed for other alternative exons such as the 86nt-long exon 3 of the *Prmt5* gene, which is inhibited by G-tracts between the polypyrimidine tract and the 3′ AG (REPAG) [29,30]. Together, such splice site-specific accumulation of reads within genes also supports the origin of the RNA-Seq reads from pre-mRNA instead of contaminated DNA. 

Therefore, the accumulated reads at the intron start or end (RISE) likely reflect pre-mRNA intermediates of alternative and/or depolarization-regulated splicing in the presence of spliced introns of the same pre-mRNA transcripts.

### 2.2. A Group of RISE Are Associated with Depolarization-Regulated Alternative Exons

**Figure 3:**
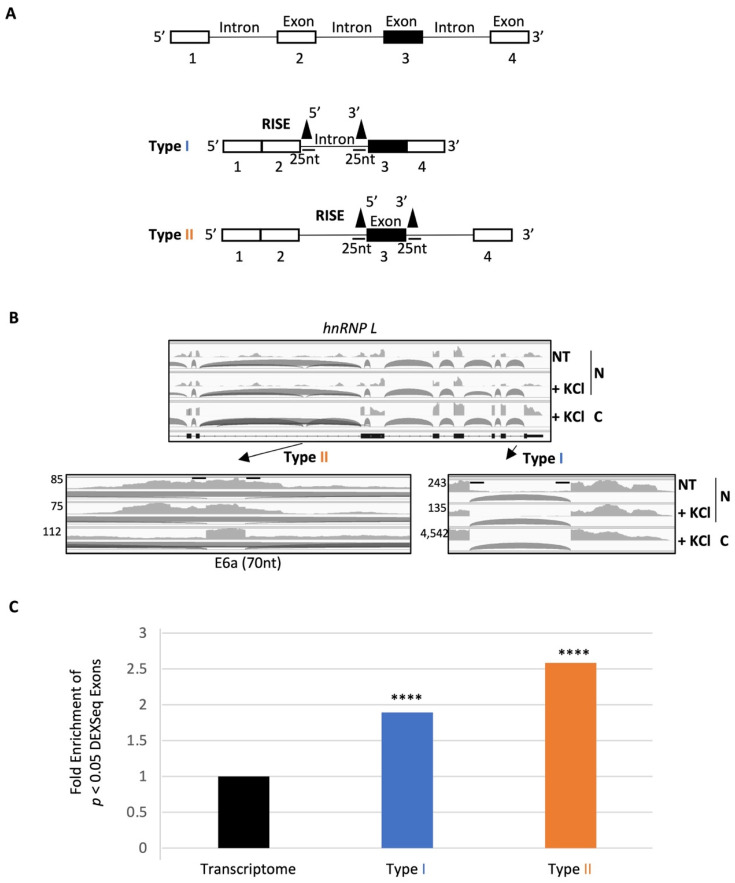
Two types of RISE pairings and their association with depolarization-regulated exons. (**A**) Diagram of RISE pairings across the same intron or exon. Arrowheads: where RISE observed. The horizontal bars represent the typical RISE length used in the analysis. Black boxes: alternative exons, White boxes: constitutive exons already spliced in most pre-mRNA intermediates as deduced by the absence of RISE. (**B**) An example of a pre-mRNA region containing both types of the RISE pairings: an IGV local view of the *hnRNP L* on chromosome 1 and enlarged regions of the RISE pairings across introns (I) or exons (II). (**C**) Fold enrichment of the percentages of depolarization-regulated exons (*p* < 0.05) among either or both types of RISE pairings over that of all such exons (2.9%) detected by DEXSeq analysis of the total RNA (total *n* = 116,950 exons/bins). ****: *p* < 2 × 10^−8^, hypergeometric density test.

Further manual examination of a group of RISE-containing splice sites of cassette exons in the IGV identified three types of such reads (Figure 3A): in the same intron (type I), across the same exon (type II), or neither type I nor II (III). We reckoned that type III perhaps was a variation of the first two in more complex situations such as pairing with farther away splice sites. Therefore, we focused on types I and II in this study. For example, for the alternative exon 6a of the *hnRNP L* gene [31], it has high levels of type II RISE across the exon in the nuclei, compared to that in the cytoplasm (Figure 3B). A pair of type I RISE is also visible in the last intron in the NT nuclear RNA with little change by depolarization, unlike the STREX (Figure 2).

To determine whether the RISE are globally enriched for depolarization-regulated exons, we filtered for the depolarization-changed ones (*p* < 0.05, KCl vs. NT) that had adjacent RISE, from DEXSeq analysis of poly(A) RNA. Exons adjacent to either type I or II RISE were enriched of changed ones by about 2 folds (Figure 3C, increased from 2.9% to 5.4% and 7.3%, respectively, or 5.5% of all 9751 RISE-adjacent exons). 

The RISE-adjacent, regulated exons comprise about 15% of 3492 depolarization-regulated exons. The remaining 2504 were detected but their adjacent RISE were below the threshold, suggesting that the splicing or regulation of most of these exons was already finished without detectable splicing intermediates 6h after depolarization. Therefore, this RISE analysis covers the group of depolarization-regulated exons that still had detectable intermediates at the 6 h time point. More exons could be covered by the analysis in future studies at earlier time points.

### 2.3. Correlation of RISE Abundance between Splice Sites across Introns or Exons in the Transcriptome

To determine if the RISE abundance is globally correlated between the 5′SS and 3′SS pairs across introns or exons, as predicted by the SS pairing in intron or exon definition (Figure 2 and Figure 3), we obtained reads typically within 25nt at the start or end of introns from an analysis of unannotated regions by edgeR (see Methods). The RISE abundance (index) was calculated by dividing its read average per nucleotide by that of the adjacent exon. The indexes increased from 0.19 (±0.03) for the 5′ 40% of detected exons of genes to 0.44 (±0.05) for the 3′ 20% of the exons (mean ± SEM, *p* < 0.0001, *n* = 74, 58 SS, respectively), consistent with the overall 5′ to 3′ direction of intron removal from nascent pre-mRNA transcripts [8,9]. In total, we obtained 985 type I (61%) and 633 type II (39%) pairs across introns or exons from 6,132 3′SS and 5,062 5′SS RISE indexes (Supplementary Figure S1), which did not correlate when they were not selected in pairs (Supplementary Figure S2).

**Figure 4:**
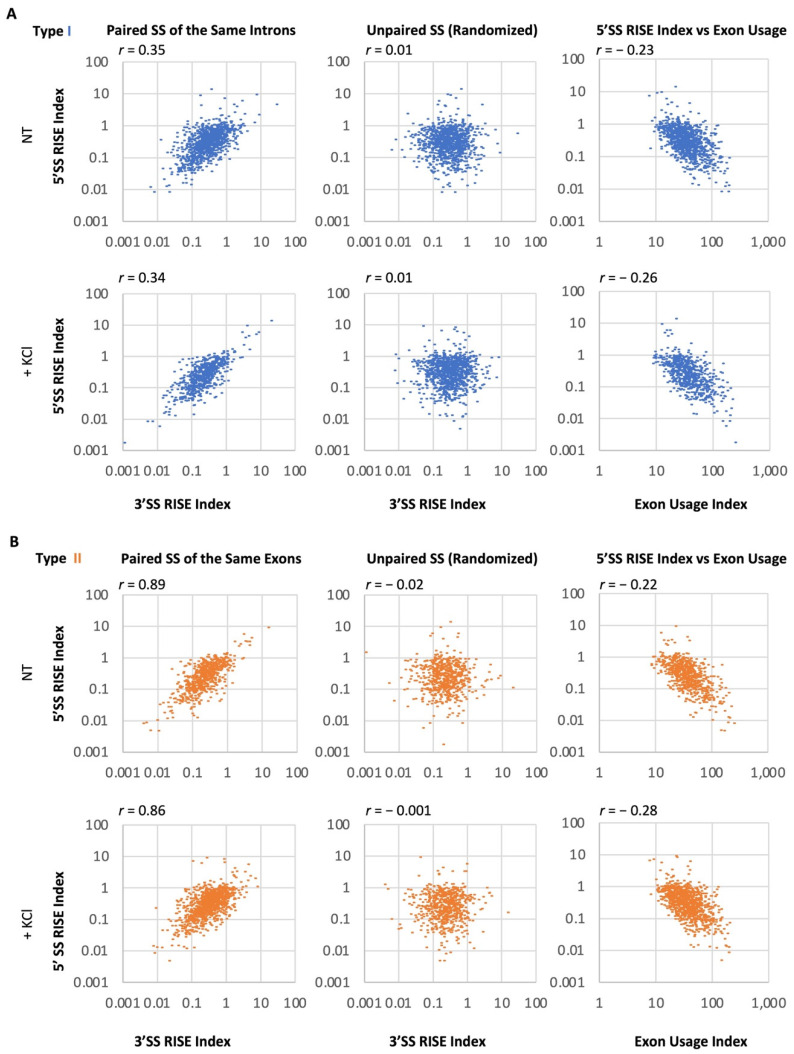
Transcriptome-wide correlation of RISE indexes across introns or exons. (**A**) Scatter plots of the 5′ and 3′SS RISE indexes (Log10) across introns (**left** panel), randomized (**middle**), or of 5′SS RISE index versus the usage of adjacent exons (**right**), in NT (**upper**) or KCl-treated (**lower**) cells. *n* = 985 type I RISE. (**B**) Similarly, as in A, except across exons. *n* = 633 type II RISE. *r*: Pearson coefficient. Each index value is the mean of three samples.

Interestingly, the paired RISE indexes overall correlated positively between the 5′SS and 3′SS across introns in the NT sample (Figure 4A, type I, Upper Left), in comparison with their unpaired SS (randomized, Figure 4A, Upper Middle). In contrast, the 5′SS RISE index inversely correlated with the usage of adjacent exons (Figure 4A, Upper Right) and so was the 3′SS RISE index (*r* = −0.18). Furthermore, similar correlation patterns were also observed in the KCl-treated samples (Figure 4A, Lower panel).

For the RISE indexes across exons, they also correlated positively, with even much higher coefficients (Figure 4B, type II, Left), in comparison with the unpaired SS (randomized, Figure 4B, Middle). In contrast, the RISE indexes were also inversely correlated with exon usage (Figure 4B, Right).

To make sure that the global positive correlation is not simply due to that the paired RISE are in the same gene transcripts, we further examined the Pearson coefficients with 218 RISE pairs of 25 genes. The mean *r* value of paired indexes is significantly higher than the randomized ones within the same genes (Mean = 0.70 vs. 0.57, *p* < 0.001, *n* = 4 groups). The stronger correlation between paired RISE indexes supports the existence of a constraint beyond their mere presence in the same genes/transcripts, which is likely the driving force for splice site pairing in intron or exon definition.

### 2.4. Global Modulation of the RISE Indexes and Their 5′SS/3′SS Index Ratios by Depolarization

**Figure 5:**
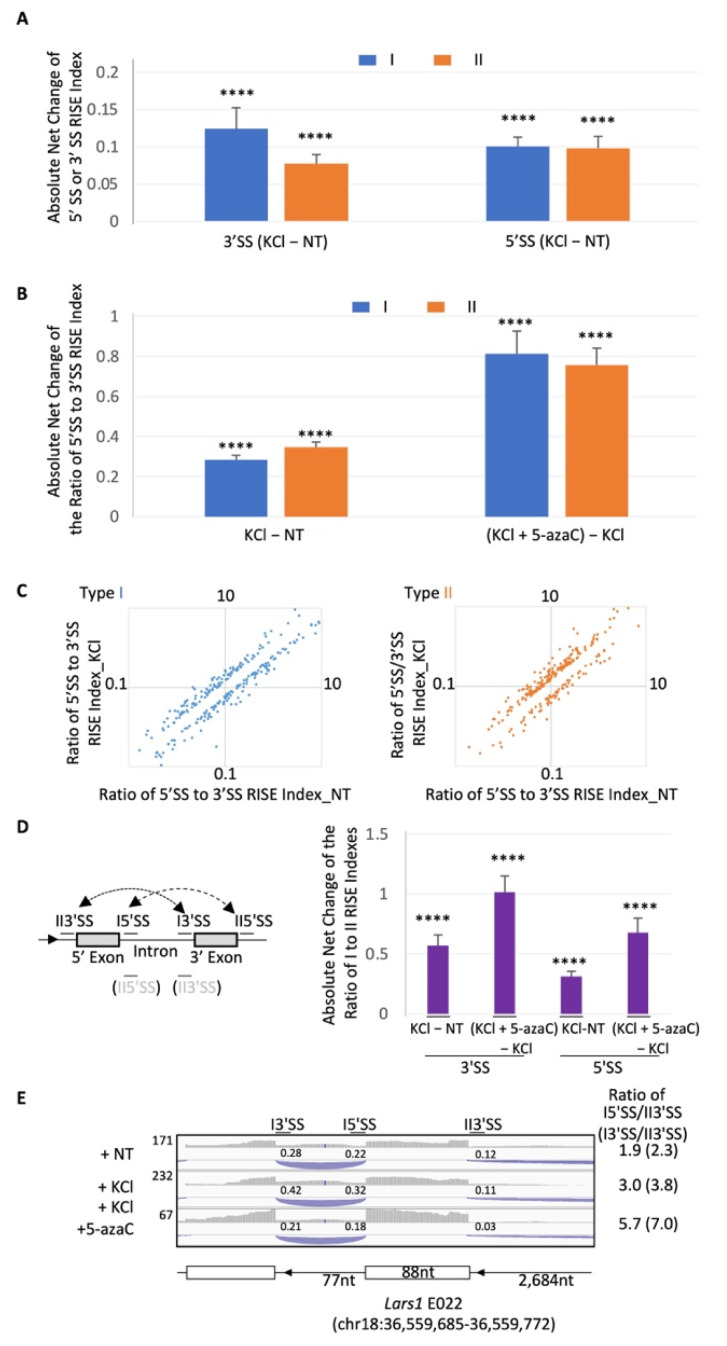
Global changes of the RISE indexes and 5′SS/3′SS RISE index ratios by depolarization. (**A**) Absolute net changes (Mean ± SEM) of the indexes of 5′ or 3′SS RISE across introns (I, *n* = 803 pairs) or exons (II, *n* = 535 pairs) by KCl versus those in NT. (**B**) Absolute changes (Mean ± SEM) of the ratios of 5′ versus 3′SS RISE indexes. (**C**) Scatter plots (Log10) of the 5′SS/3′SS ratio changes of RISE pairs by KCl, by more than 20% of the NT samples in (**B**) (*n* = 266 and 254 pairs for types I and II, respectively). (**D**) RISE index shift between the type I and II by KCl. Left, a diagram of an example of type I mixed with either a 5′ or 3′ type II RISE pairing, with the splice sites labeled as in the graph to the right. Note that the I5′SS or I3′SS, thus labeled after type I SS for simplicity, can also be type II 5′SS or 3′SS (in grey), respectively. The dash lines with arrows denote the shift directions, which could be either way by KCl (vs NT) or 5azaC (5azaC + KCl vs. KCl). Arrowhead: gene direction. Right, absolute net changes (Mean ± SEM) of the I3′SS/II3′SS or I5′SS/II5′SS RISE ratios when the 5′ or 3′ exon is involved in both types I and II, respectively. *n* = 178, 178, 147 and 147, from left to right. (**E**) An example of the effect on the RISE indexes by KCl-depolarization and 5-azaC for those in (**B**) and (**C**): Diagram of the *Lars1* exon E022 and its downstream intron with both types I and II RISE pairing, with indicated average indexes of RISE as well as their index ratios of I5′SS/II3′SS (type I pairing RISE) or I3′SS/II3′SS (type I or II RISE). Arrowheads on introns: gene direction. ****: *p* < 0.0001, one sample *t*-test of the population Mean value of net changes against the hypothesized value of 0 (no net changes).

To determine the global effect by depolarization, we examined the index net changes and the ratios of the 5′SS to 3′SS RISE indexes (Figure 5, statistics by one sample *t*-test [32]). Globally, depolarization caused a significant shift from the NT indexes (Figure 5A) as well as the ratios of the 5′SS to 3′SS indexes (Figure 5B,C), as measured by the absolute net changes. Moreover, the depolarization effect was disrupted by 5-azaC (Figure 5B), an inhibitor of DNA methylation likely controlling alternative splicing through DNA binding proteins and associated splicing factors [33,34,35,36]. 

We also examined the depolarization effect on the shift of RISE indexes between type I and II pairs by measuring their ratio changes between competing splice sites (Figure 5D), as expected in the shift between exon and intron definitions [9,37]. On average, the ratio of the competing 3′ or 5′SS RISE indexes was significantly shifted by depolarization and further by 5-azaC, as measured by the absolute net changes. For example, the ratio of the RISE indexes across the *Lars1* exon E022 was increased by depolarization and further by 5-azaC; as is the ratio between the competing 3′SS (Figure 5E). This is consistent with a shift from type II to type I RISE pairing, i.e., from delayed exon to intron definition. 

Together, these observations support that depolarization globally modulates the RISE indexes and splice site pairing in intron/exon definition or its transition, which is probably disruptible by DNA methylation changes.

### 2.5. RISE Are Associated with Weak Splice Sites and Short Introns or Exons

**Figure 6:**
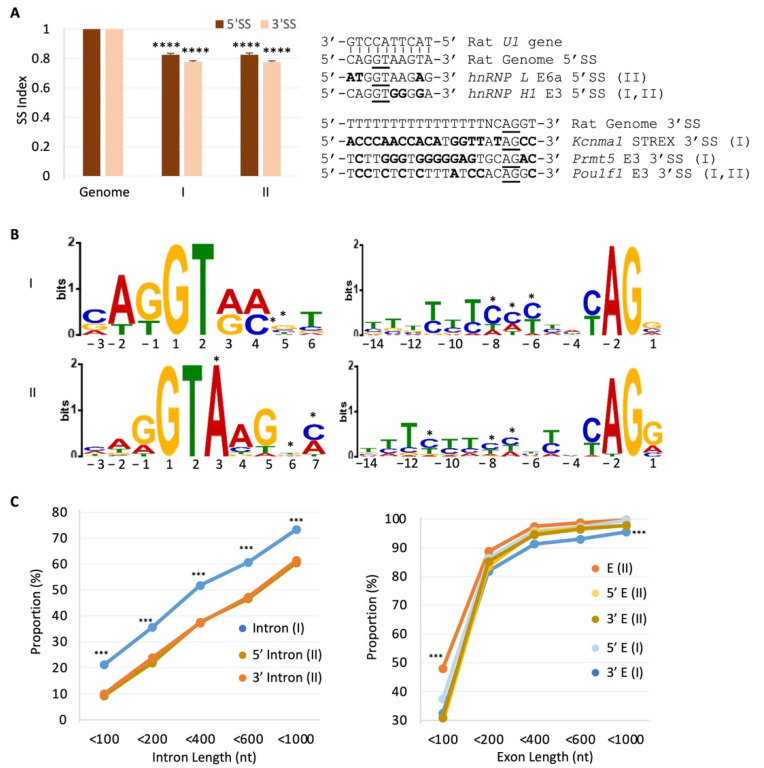
Properties of the RISE-associated splice sites, exons, and introns. (**A**) Indexes of the nucleotide matrices of the involved splice sites. Left: bar graphs of the splice site indexes of the most abundant nucleotides of the consensus splice sites of *Rattus norvegicus* (index = 1, Ref. [34], Nguyen and Xie, *Gene*’18, using Ensembl Genome Metazoa Release 96), and the RISE-associated splice sites (−3–+10 for the 5′SS, and −20–+2 for the 3′SS, Mean ± SEM, *n* = 186,851, 188,528, 67, 44, 48, 48 splice sites, from left to right). ****: *p* < 0.0001, one sample *t*-test compared to the genome. Right: examples of the RISE-associated 5′ or 3′SS sequences. For the 5′SS, the genome consensus matches perfectly with the complementary consensus region of 81 *U1* snRNA sequences from rats. Nucleotides different from the genome consensus are in bold and the 5′ GT and 3′ AG are underlined. Bracketed are the associated types of RISE pairings. (**B**) Logo representation of the information content of the enriched nucleotides of the 5′ (Left) and 3′ (Right) splice sites with either type of the RISE pairing and DEXSeq *p* < 0.05, in comparison with the SS consensus sequences of the rat genome in (**A**) (Right panel). *n* = 28 (I, 5′SS), 50 (I, 3′SS), 34 (II, 5′SS), 34 (II, 3′SS) splice sites, and *E*-value < 1.3 × 10^−28^ and 5.6 × 10^−42^, for the 5′ and 3′SS, respectively. *: nucleotides deviated from the species consensus. (**C**) Percent distribution of intron/exon lengths. Left: intron length distribution. ***: *p* < 6.7 × 10^−10^, hypergeometric density test compared to either 5′ or 3′ introns of the exons flanked by type II RISE pairings. Right panel: similarly as in the left, but for exon lengths. ***: *p* < 4.3 × 10^−6^, E (II) in comparison with the 5′ or 3′ E (I). E: exon. *n* = 644 or 311 introns/exons, for each type I or II data point (non-overlapping), respectively.

Alternative splicing tends to be delayed or less efficient during co-transcriptional splicing [9,10]. To determine if the RISE-associated splice sites are particularly weak in general to delay their usage, we compared their nucleotide matrices with the consensus motifs of the over 180 thousand splice sites in the annotated rat genome [38], which reverse complements perfectly with the 5′SS-binding motif of the rat *U1* snRNA (Figure 6A). The matric indexes of the associated splice sites of type I and II RISE are similar (*p* > 0.8). However, they deviate significantly from the genome-wide consensus (Figure 6A). For instance, the 5′SS of the hnRNP L E6a and hnRNP H E3, and the 3′SS of the STREX, PRMT5 E3, and Pou1f1 (Pit1) E3, all contain multiple nucleotides different from the consensus. 

Further analysis of the splice sites of the RISE-adjacent, depolarization-regulated exons using multiple Em for motif elicitation (MEME) [39], showed distinctly enriched nucleotides at both the 5′ and 3′ splice sites (Figure 6B, asterisks). Such deviations of the nucleotides from the species consensus could even generate splicing regulatory elements besides weakening the SS strength. For example, the enriched C4 and C7 of the 5′SS mismatch with the U1 snRNA, and the 3′SS CA dinucleotide repeats in the 3′SS of the *Pou1f1* (*Pit1*) E3 and STREX are targets of splicing regulation by hnRNP L/LL upon depolarization and calcium signaling [14,15]. 

We also compared the lengths of the introns or exons associated with the two types of RISE pairs. The type I RISE indexes did not correlate with intron lengths overall (*r* = −0.005 on average), but the type II inversely correlated with exon lengths (*r* = −0.37 on average, for <400 nt exons), consistent with a higher index for delayed exon definition of shorter exons. More interestingly, the type I and II groups showed significant differences. Type I is associated with more of the introns less than 1000 nt, compared to type II (Figure 6C, Left, 72% and 60%, respectively), though both are less than around 3000 nt in the length of the majority of rat introns [4]. Moreover, type II is associated with more short exons (Figure 6C, right), in particular, the less-than-100 nt ones, compared to their immediate 5′ or 3′ exons of the same genes or to the type I-associated ones. In contrast, the distribution of the type I exon sizes is similar to that of the rat genome. 

Together, the RISE are associated with weak splice sites, shorter introns (more so in type I) or exons (type II) in the rat genome, consistent with their inverse correlation with exon usage (Figure 4) and modulation by depolarization and 5-azaC (Figure 5).

### 2.6. The RISE Are Highly Associated with Genes in Basic Cellular Processes or in Hormone Production

Although the RISE indexes are inversely correlated with exon usage and globally modulated by depolarization (Figure 4 and Figure 5), after splicing, only a small percentage of the associated exons ended up exhibiting significant changes by depolarization (Figure 3C and text). This divides the exons into two groups: ones that are alternatively spliced but not regulated by depolarization sufficiently to change their exon levels (E6a in Figure 3B), and ones that are both alternatively spliced and regulated by depolarization to change their exon levels (STREX [13], Figure 2A,B). We thus examined the potential gene clusters of the two groups separately to see if different cellular functions are targeted.

**Figure 7:**
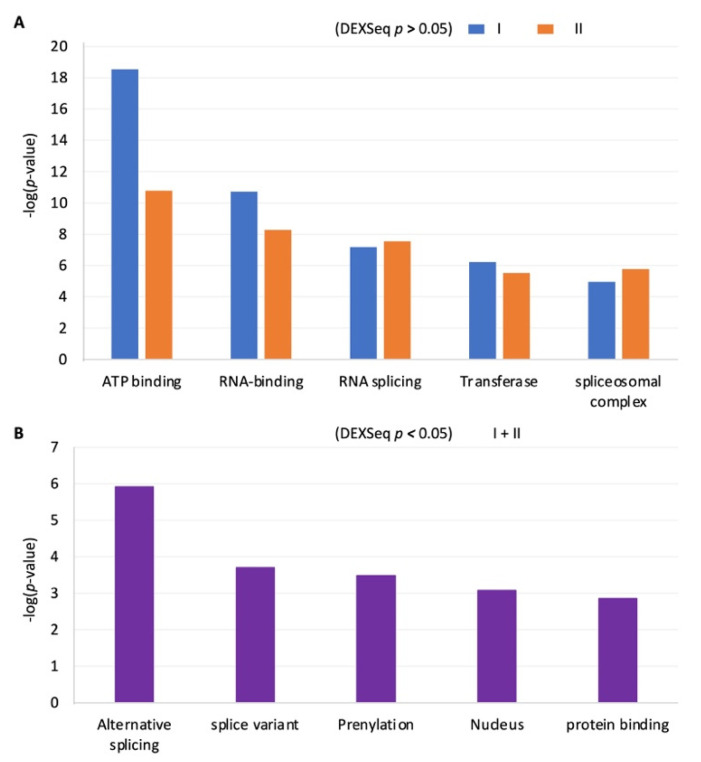
Significantly enriched functional clusters of the genes identified with RISE. Bar graphs of the –LOG (*p*-value) of the DAVID functional clustering analysis for genes with type I or II RISE pairing of adjacent exons without significant changes by depolarization ((**A**), *p* < 0.05 by DEXSeq, *n* = 663 and 456 gene IDs for type I and II, respectively) and those with adjacent exons changed significantly by depolarization ((**B**), *p* < 0.05 by DEXSeq, *n* = 34 gene IDs).

DAVID functional clustering analysis of genes whose RISE-associated exons were not changed upon depolarization (*p* > 0.05 by DEXSeq) gave rise to clusters for basic cellular processes such as signaling (ATP-binding or transferases) or RNA splicing (Figure 7A). The top clusters are the same for the type I and II groups. However, interestingly, analysis of genes whose RISE-associated exons were changed significantly by depolarization (*p* < 0.05 by DEXSeq) did not give rise to the same clusters. Instead, it gave rise to clusters for more specific pituitary functions: genes with alternative splicing, prenylation, nuclear functions, and protein interactions (Figure 7B). For example, the “alternative splicing” cluster includes the pituitary-specific transcription factor *Pou1f1* (*Pit1,* see also Figure 6A), essential for pituitary cell differentiation and transcription of prolactin and growth hormone [40,41], and *Sequestosome 1* (*p62*), essential for luteinizing hormone production [42], as well as the *cullin-associated and neddylation-dissociated 2* (*Cand2*) with a homolog *Cand1* regulating vasopressin secretion [43]. Thus, the detected RISE-associated genes cluster for either basic or tissue-specific cellular functions depending on their regulation status by depolarization.

## 3. Discussion

In this report, we analyzed the RISE of the nuclear RNA-Seq data together with those from cytoplasmic or whole cell RNA to identify the delayed pairing patterns of splice sites in depolarization-regulated splicing. Their splice site-specific accumulation within genes or around exons and their regulation by depolarization/5-azaC support the pre-mRNA origin of the reads rather than genomic DNA, where in the latter case, random distribution of reads and non-regulation is expected. The analysis has led to the first-time observation of not only delayed 3′ splice site usage of the endogenous STREX and other exons we have studied (Figure 2) but also globally delayed, or depolarization-regulated, across intron- or exon-pairing of splice sites in the pituitary cells. 

These introns or exons in general tend to be shorter in the respective pairing groups (Figure 6). However, individual cases may vary greatly. For example, the type I RISE pair’s introns are mostly less than 1kb in the rat cells (Figure 6C), but the STREX upstream intron is close to 10 kb (Figure 2). The long lengths are in contrast to the mostly less-than-0.1kb of intron lengths for intron definition in yeast, worm, or arthropods [2,3,4,8,10]. An explanation for the SS pairing or definition of such long introns perhaps could be helped by the reported 5′SS and 3′SS interactions or recursive splicing [1,5,6,7,44,45,46]. Moreover, the type II RISE pair’s exons are only 124nt on average, but there are also 10 exons longer than 496 nt, at which length exon definition is abolished in vitro [1]. Of these long exons, five are penultimate exons that involve the last intron splicing, which can be influenced by polyadenylation [47,48], or 5′-to-3′ directionally delayed splicing during transcription [8,9]. However, the other five are middle exons (3rd or higher rank). How these long middle exons are recognized through across-exon pairing or whether they are exceptions awaits validation.

We detected more instances of across-intron than -exon RISE pairs, which might be just for this cell line under the experimental conditions since they represent only the delayed splice sites at the 6h time point. However, the correlation coefficients for the across-exon pairs are much higher than the across-intron ones (Figure 4). One could argue that the across-exon RISE pairs are closer to each other; therefore, their read abundance could covariate more. If so, the same should be true for the intron pairs within similar size ranges. However, we did not observe a correlation between the intron lengths and RISE indexes (*r* = −0.005 on average), even among those less than 400 nt (*r* = 0.02 on average). Alternatively, the across-intron RISE may pair with those further away rather than in the same introns only (type III), thereby adding to more variation, but the same could also be applicable to the RISE of the across-exon pairs. Therefore, the observation likely supports inherently stronger exon than intron definition in the mammalian cells.

The RISE index analysis has allowed a glimpse of a group of splicing intermediates in the transcriptome. For a more complete and dynamic view of transcriptome-wide RISE pairing and its regulation by depolarization, however, many more intermediates have to be detected at additional time points and/or with more in-depth nuclear RNA-Seq.

Potential molecular mechanisms for the regulation of splice site pairings in these cells by KCl depolarization could involve not only the previously identified hnRNP L/LL at the 3′SS of STREX [15,18] but also other partners that allow the 3′SS to cross-talk with the upstream 5′SS. These could include SR proteins, U snRNP components, or *cis*-acting pre-mRNA sequence motifs as demonstrated previously in other systems [1,5,6,7]. For the 5-azaC effect on the splice site pairings, the cytidine analog has been known to regulate alternative splicing [49]. However, its underlying molecular mechanism is unclear. Its inhibition of DNA methyltransferases and thereby DNA methylation perhaps affects the binding of protein factors including CTCF and MeCP2 [33,36], which could regulate splicing through interaction with splicing factors such as YB-1 [35], or histone deacetylase [34]. Particularly relevant is that the exon CpG DNA methylation level is higher than the flanking introns likely facilitating the exon definition [34,50]. Alternatively, 5-azaC’s other effects on splicing through DNA methylation could involve kinetically the rate of transcription or RNA polymerase II pausing [51,52].

In summary, the nuclear RNA-Seq analysis of intron RISE has allowed us to see for the first time the coordinated transcriptome-wide splice site pairing among endogenous pre-mRNA transcripts that is consistent with delayed intron and/or exon definition. The global view suggests that there is an overall much stronger exon than intron definition in the mammalian transcriptome. The detected intermediates of the weak splice sites and short introns/exons likely impact genes in basic as well as pituitary-specific cellular functions depending on their regulation by depolarization.

## 4. Materials and Methods

**Cell culture:** We cultured rat GH_3_ pituitary cells in Ham’s F10 nutrient mixture with 10% horse serum plus 5% fetal bovine serum with 5% CO_2_ at 37 °C.

**KCl treatments, total or cytoplasmic RNA extraction, and RNA-Seq analysis:** For treatment, KCl (50 mM) was added and incubated for 6h before RNA extraction. We extracted total RNA with the GenElute™ Mammalian Total RNA Miniprep Kit (#RTN350-1KT, Sigma Aldrich, St. Louis, MO, USA). We extracted cytoplasmic RNA using our previous nucleo-cytoplasmic fractionation protocol [12,53], the samples of which were from an adaptive splicing experiment where the cells were also KCl-treated but for 6 times with 18h intervals. The multiple KCl treatments did not affect the main nuclear or cytoplasmic localizations of the mRNAs tested.

RNA-Seq was carried out after purification of poly(A) RNA using oligo d(T) and random-primed for cDNA synthesis following the protocol of Illumina Truseq stranded mRNA sample preparation. Illumina HiSeq4000 paired-end 100-bp sequencing was used for the total RNA of non-treated (NT) and KCl-treated (6 h) samples, and the Illumina NovaSeq 6000 S2 paired-end 100-bp sequencing was used for the cytoplasmic RNA samples. RNA-Seq analyses by edgeR or DEXSeq were performed as in our previous report [12].

**Nuclear RNA preparation and RNA-Seq analysis:** The nuclear RNA samples were obtained from a test intended for lariat and RNA secondary structures. Briefly, GH3 cells were untreated, treated with KCl (50 mM), or KCl (50 mM) plus 5-azaC (50 μM), respectively in triplicate (three wells of cells). Upon 6h treatment, cells were collected and nuclear RNA extracted in a procedure modified from the nucleo-cytoplasmic fractionation protocol [12,53]: cells were lysed with cold 0.325% NP-40 buffer (10 mM Tris-Cl pH 7.5, 75 mM NaCl, 0.325% IGEPAL CA-630), centrifuged, and the nuclear pellet washed with ice-cold PBS plus 1 mM EDTA, resuspended and lysed in 0.65% NP40 buffer (10 mM Tris-Cl pH 7.5, 75 mM NaCl, 0.65% IGEPAL CA-630, and 10mM FeCl3 [54,55]) with 10 µg/mL psoralen [56], chilled in an aluminum block on ice for 10min, and ultraviolet-irradiated (365 nm) for 20min., and extracted with the QIAquick Gel Extraction Kit (Qiagen, Cat.# 28706, Hilden, Germany) for nuclear RNA.

Approximately 1 μg of nuclear RNA from each sample of the three groups was reverse transcribed using random primers for NEB rRNA-depleted (HMR) stranded library and NovaSeq 6000 sequencing. On average, 42 million uniquely mapped reads (paired, ±6.6, *n* = 12) were obtained for each sample. 

For the RISE analysis, the following criteria were empirically chosen after referring to a similar method in the previous study of 3′SS [8], and testing different exon levels or intron bin lengths: more than 8 raw read counts on average per nucleotide position mapped to the 25 nt bins at the intron start/end. Some terminal bins (48%) of introns were kept between 26–37 nt inclusive in cases less than 25nt left when binning from the 5′ toward the 3′ end of the plus (+) strand. Subsequent analysis criteria include introns longer than 50 nt, exons longer than 12nt, and corresponding exon base means more than 20 by DEXSeq analysis of whole cell poly(A) RNA. RISE indexes of the 3′ terminal intron, which may involve polyadenylation [8,48,57,58], showed no apparent difference in pairing correlation from the transcriptome-wide population and were thus included in the analysis. The reference rat genome used is rat RN6. The data for the figures are in Supplementary Tables S1–S7.

**Statistical tests:** All samples were in triplicate. Two-tailed Student’s *t*-test was used unless otherwise noted. The DEXSeq and edgeR analysis use Fisher’s exact test [59,60].

## Data Availability

Data supporting the results are available in the supplementary materials.

## Supplementary Materials

The supporting information can be downloaded at: https://www.mdpi.com/article/10.3390/ijms231710157/s1.
