# Supplementary material for: Transcriptome-Wide Detection of Intron/Exon Definition in the Endogenous Pre-mRNA Transcripts of Mammalian Cells and Its Regulation by Depolarization
Source: Int J Mol Sci. 2022 Sep 5;23(17):10157. doi: 10.3390/ijms231710157 (PMC9456152; doi:10.3390/ijms231710157)
Supplement: Supplementary file 1 [file ijms-23-10157-s001.zip › ijms-1887187-supplementary/ijms-1887187-supplementaryfigures.pdf]

Supplementary Figure S1

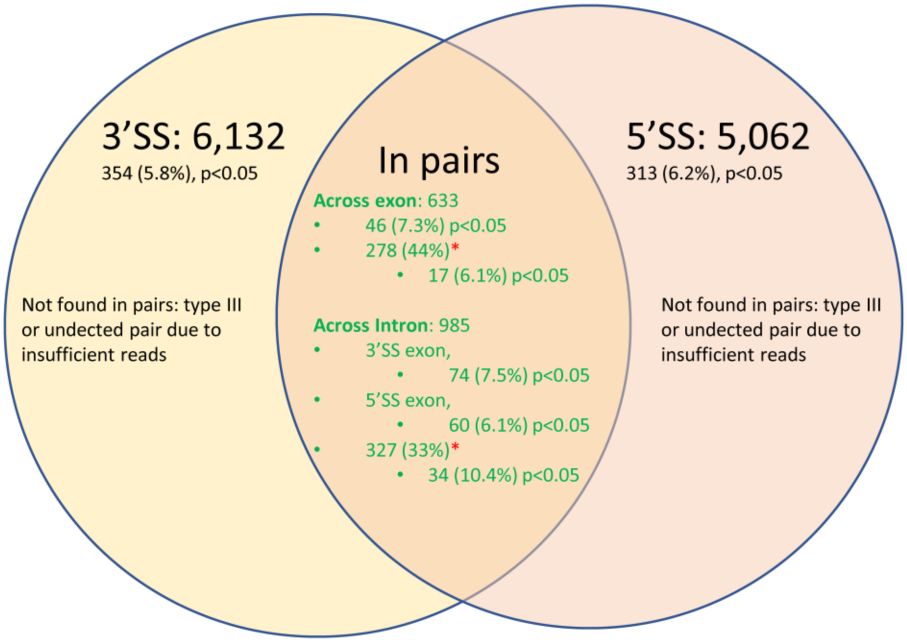

Figure S1. Distribution of RISE indexes obtained from the RNA-Seq data. p-values are from the DEXSeq for exon changes by KCl depolarization. *: >20% change of the ratio of 5SS/3SS RISE index by KCl.

Supplementary Figure S2

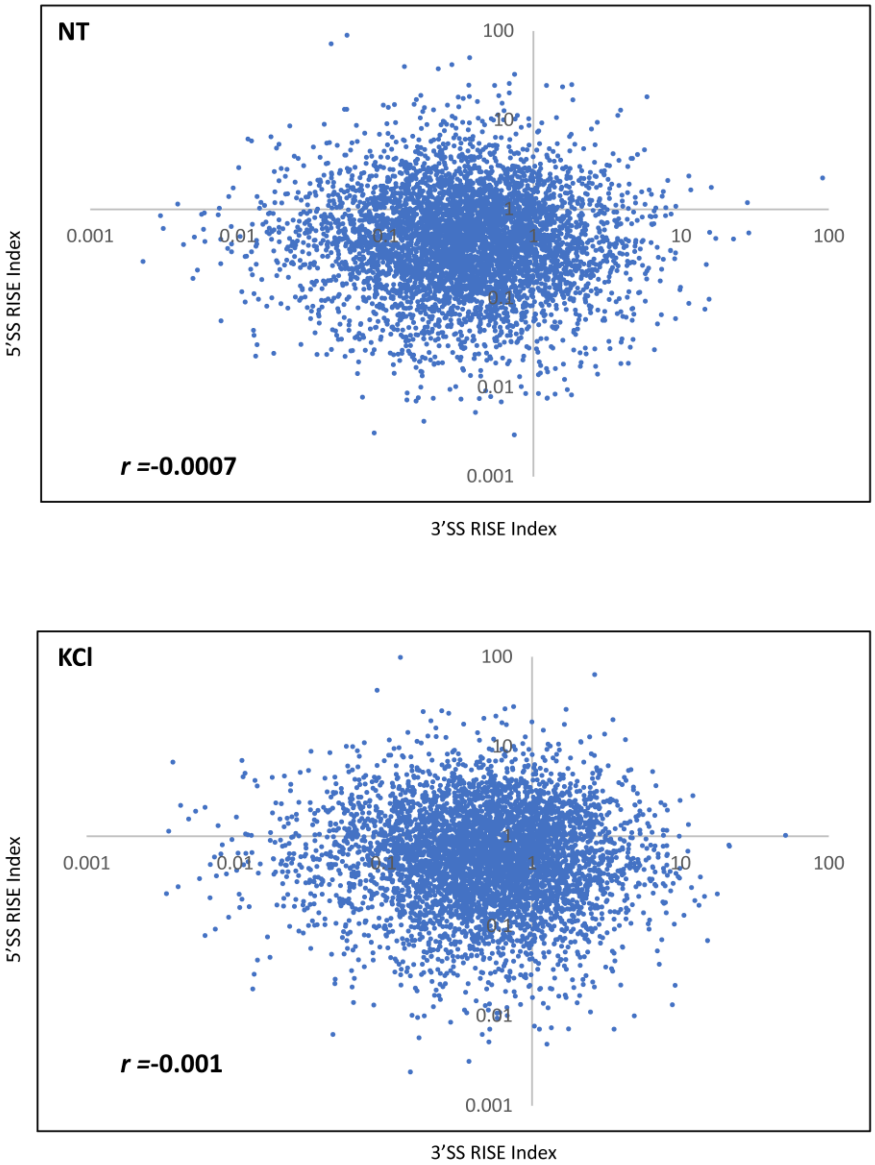

Figure S2. Distribution pattern of 5,062 RISE indexes of 5'SS and 3'SS unselected in pairs from the nuclear RNA-Seq data (see also **Figure S1**).
